# A Novel and Low-cost Approach for Intravitreal Injection in an Experimental Model of Endophthalmitis

**DOI:** 10.18502/jovr.v18i3.13775

**Published:** 2023-07-28

**Authors:** Dhanwini Rudraprasad, Jaishree Gandhi, Poonam Naik, Milind N Naik, Chenchu Naidu, Dilip Kumar Mishra, Joveeta Joseph

**Affiliations:** ^1^Jhaveri Microbiology Centre, Brien Holden Eye Research Centre, LV Prasad Eye Institute, Hyderabad, Telangana, India; ^2^Manipal Academy of Higher Education, Manipal, Karnataka, India; ^3^Ophthalmic Plastic Surgery & Facial Aesthetics, LV Prasad Eye Institute, Hyderabad, India; ^4^Ophthalmic pathology Laboratory, LV Prasad Eye Institute, Hyderabad, Telangana, India

**Keywords:** Endophthalmitis, Histopathology, Infection, Mice Model, Pathobiology

## Abstract

**Purpose:**

Animal models are necessary in understanding the pathogenesis of endophthalmitis and are also necessary to assist the development of new therapeutics for this sight-threatening ocular inflammation. Hamilton syringes are usually preferred to inject pathogens when performing experiments on test subjects, however, this method has technical and financial disadvantages. In this study, we report the findings and assess the related benefits of applying a novel low-cost intravitreal injection technique to initiate endophthalmitis in a mouse model while using the Eppendorf tip and a 26G needle.

**Methods:**

The 18-hr culture of clinical isolates of bacteria (*Staphylococcus aureus* and *Pseudomonas aeruginosa*) and fungus (*Aspergillus flavus* and *Candida albicans*) were resuspended to a final concentration of 10,000 colony forming units (CFU)/1 µL which were separately injected intravitreally into C57BL/6 mice (6–8 weeks) using a 0.1–2.5µL pipette attached to the modified Eppendorf tip with a 26G needle. The contralateral eye served as vehicle/uninjected control. Disease progression was determined by assessing the corneal haze, opacity, bacterial burden, and retinal histology of the eyes used in the model. Following euthanization, bacteria-infected mice were enucleated after 24 hr of the initial injection, and fungus-infected mice after 72 hr.

**Results:**

Of the 50 mice injected, the modified technique was successful in 48 mice. Two mice were excluded due to cataract formed by accidental injury to the lens. The experimental endophthalmitis mice model successfully mimicked the natural clinical course. Clinical assessment and histopathology confirmed the influx of inflammatory cells into the posterior segment of the eye along with dissolution of retinal architecture.

**Conclusion:**

Our novel method of injection using a modified Eppendorf tip and 26G needle yielded a cost-effective mouse model of clinical endophthalmitis, resulting in reproducible infection for understanding various aspects of its pathobiology.

**Figure 1:**
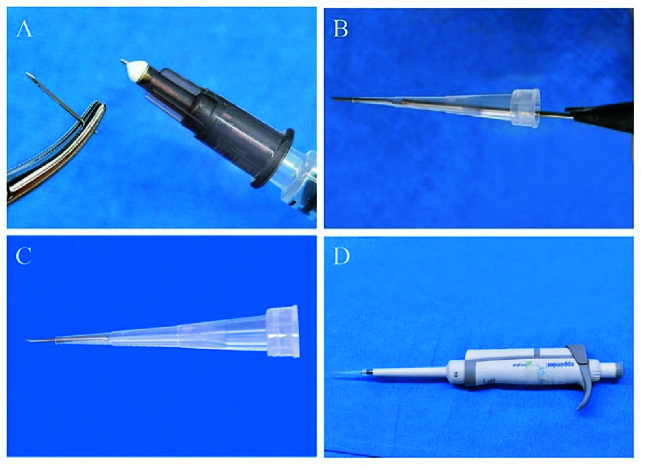
Preparation of injection equipment. (A) The needle from 1 mL syringe was detached from the needle hub. (B & C) Needle was inserted into 10 µL Eppendorf tip and the length was adjusted with a dissection needle by gently pushing the needle forward. (D) Pipette attached to the tip with needle.

**Figure 2:**
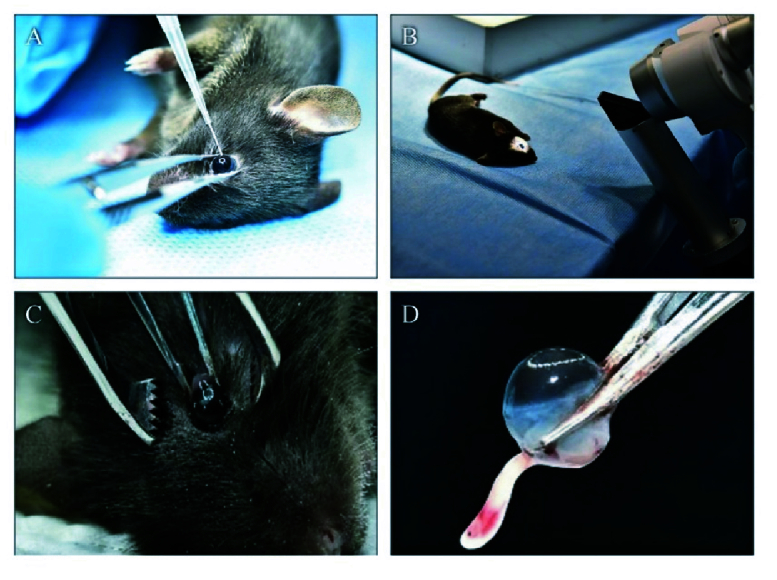
Infection and clinical scoring. (A) Forceps were used to protrude the eyeball and expose the limbal region. (B) Needle was inserted intravitreally through posterior of limbus and 1 µL of bacterial (10,000 CFU)/fungal (15,000 spores/cells) suspension was injected. (C) Disease severity was monitored through slit-lamp. Eyeball was protruded with serrated forceps and blunt forceps was slowly glided toward the back of the eyeball. (D) Eyeball was removed along with the optic nerve.

**Figure 3:**
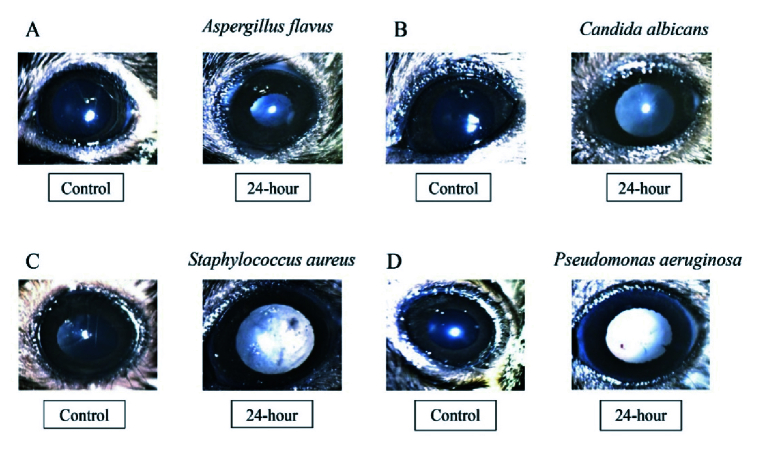
Slit-lamp images illustrating the course of disease progression. C57BL/6 mice received intravitreal injections of either sterile saline or 10000 spores of *Aspergillus flavus* (A), 10000 cells of *Candida albicans *(B), 10000 CFU of *Staphylococcus aureus *(C), and *Pseudomonas aeruginosa *(D). Infected eyes were monitored at 24-hr post-infection. Visually, the opacity of the eye increases as the disease progress with time when compared to control eye.

**Figure 4:**
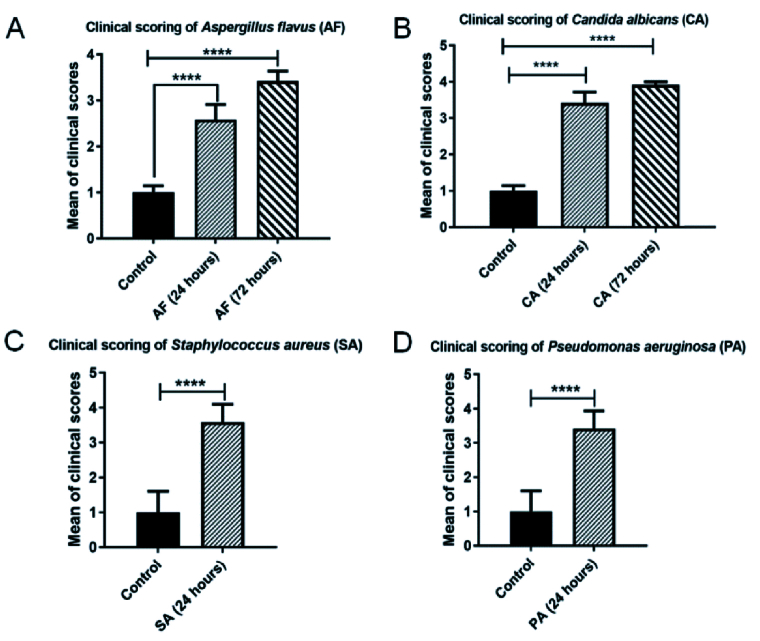
Clinical score analysis of infectious endophthalmitis. Bar graph represents the mean clinical scores of (A) *Aspergillus flavus* and (B) *Candida albicans*, (C) *Staphylococcus aureus *and (D) *Pseudomonas aeruginosa*. Fungal-infected mice eyeballs were monitored for the disease progression at 24- and 72-hr intervals, while bacterial-infected eyes were monitored at 24 hr. The eyes were clinically scored from 0 to 4 by a masked ophthalmologist according to the disease severity. Infected eyes showed significant increase in clinical scoring when compared with the control. Values represent mean ± SEM for *n* = 3 eyes per group.

**Figure 5:**
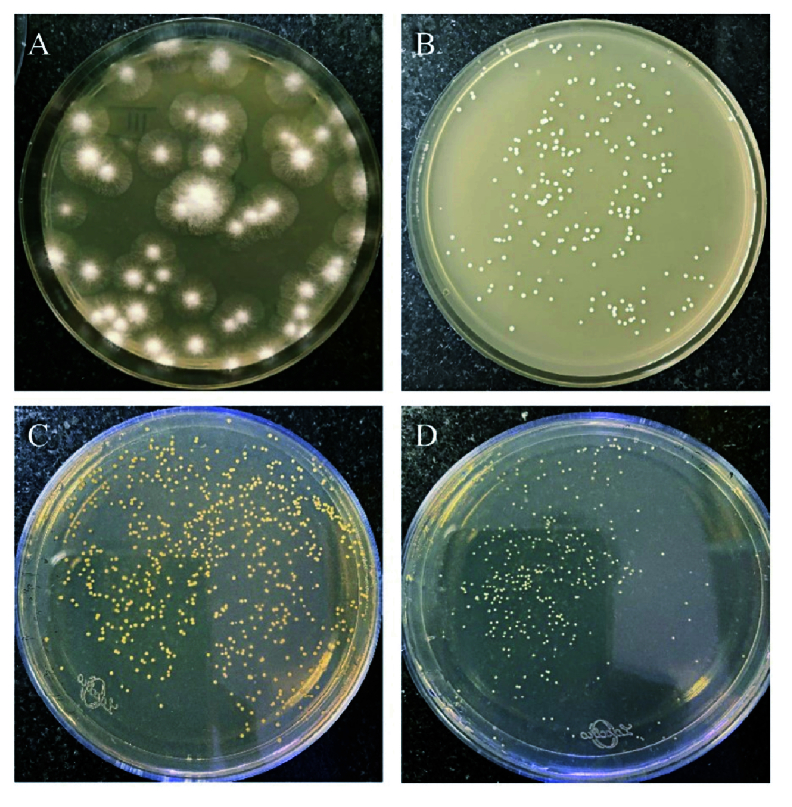
Isolated pure colonies of (A) *Aspergillus flavus*, (B) *Candida albicans*, (C) *Staphylococcus aureus*, and (D) *Pseudomonas aeruginosa* plated onto PDA/MHA from the supernatant of homogenized 24-hr-infected eyeballs.

**Figure 6:**
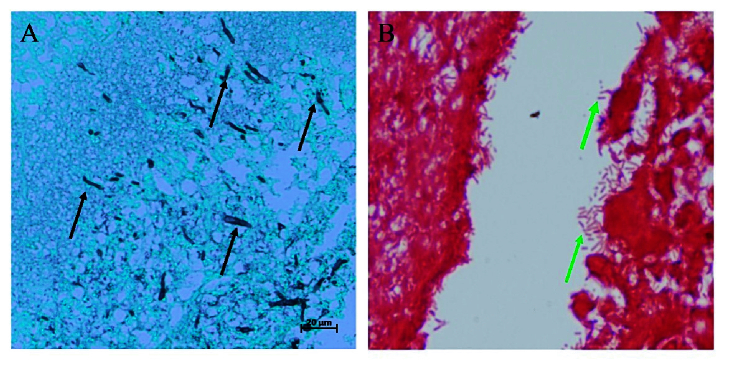
Gross-stained sections of mice eyeballs infected with (A) Fungus (*Candida albicans*) viewed under GMS stain and (B) Bacteria (*Pseudomonas aeruginosa*) viewed under grams stain. Black arrows in “A” indicate the presence of fungal filaments in the posterior segment of the eye and green arrows in “B” indicate the presence of as gram-negative bacilli in the vitreous region of the eye confirming the accurate injection site and progress of endophthalmitis.

**Figure 7:**
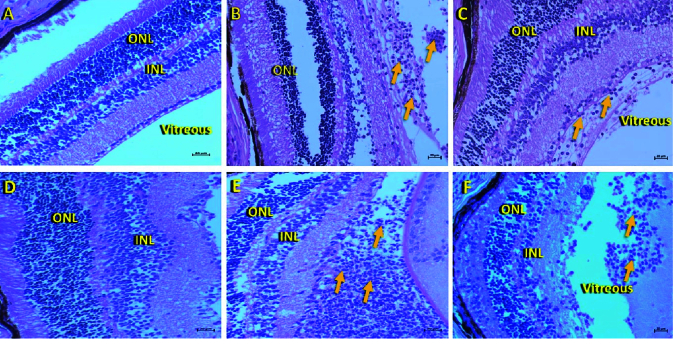
H&E-stained retinal histological sections of control and infected mice eyeballs revealed the neutrophil infiltration (indicated by orange arrows) in (B) *Aspergillus flavus*, (C) *Candida albicans*, (E)* Staphylococcus aureus*, and (F)* Pseudomonas aeruginosa*-infected eye when compared to (A & D) control (sterile 1 × PBS injected). Magnification: 40 × . ONL, outer nuclear layer; INL, inner nuclear layer

**Figure 8:**
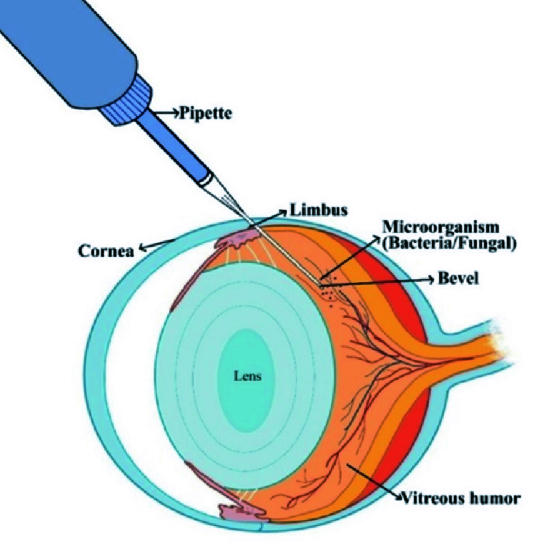
Schematic representation of a mouse intravitreal injection using a sterile needle attached to the micropipette with 1 µL fungal/bacterial suspension, which was inserted into the mid-vitreous through the posterior limbus, with bevel facing upward and held steadily before gently pressing the plunger of the pipette to release the suspension.

**Table 1:** Clinical scoring of mice model of endophthalmitis.


**Score**	**Anterior chamber**	**Red reflex**	**Vitreous**	**Retinal clarity**
0	No flare, no cells/field, no iritis	Red	Clear	Details distinct
1+	Slight flare, 1–5 cells/field, iris vessels slightly dilated	Slightly diminished	Slight haze	Details slightly obscured by infiltrate
2+	Obvious flare, 10–20 cells/field, mild dilation of iris vessels	Mildly diminished	Mild haze	Details mildly obscured by infiltrate
3+	Moderate flare, 20–50 cells/field, significant dilation of iris vessels	Moderately diminished	Dense haze	Details moderately obscured by infiltrate
4+	Intense flare, *** > *** 50 cells/field, severe iritis	No reflex (i.e., white)	Opaque vitreous	Details completely obscured by infiltrate
	
	

##  INTRODUCTION

Endophthalmitis is an intraocular infection, caused either by bacteria or fungi causing vitritis, and may occasionally result in rapid loss of vision.^[1]^ Often, it is a complication of ocular surgery or open globe injury (exogenous), but rarely due to hematogenous spread (endogenous).^[2,3,4]^ The severity of the disease depends on the inoculum size, pathogen factors, host factors, and the clinical course. Earlier we reported that 85% of endophthalmitis were bacterial, and 12% were fungal.^[5]^ While *Staphylococcus* species was the most prevalent organism, *Pseudomonas aeruginosa* was the predominant Gram-negative bacteria identified.^[5]^ Similarly, among the fungal pathogens, *Aspergillus flavus* was found to be more common.^[5]^

Experimental murine models of infection are desirable to assist in understanding the pathobiology of endophthalmitis. They allow evaluation of infection parameters, and study of new therapeutic regimens. Of the several animal models, the mice model of experimental endophthalmitis is the most successful due to the ease of availability of knock out strains and similar courses of infection as seen in human endophthalmitis.^[6,7]^ However, since the vitreous volume of the mouse eye is only 7 µL,^[8]^ only minute amounts of suspension can be injected, requiring specialized equipment and techniques to achieve reproducible infection.

A Hamilton syringe with a blunt-end 33-gauge needle is popularly used to inject bacterial/fungal suspension into the vitreous chamber of mice.^[9,10]^ Long length of the needle, blunt tip, and high cost are some of the drawbacks associated with the use of the Hamilton syringe. In this study, we illustrate a novel intravitreal injection device that is compact, effective, simple, and economical to assemble. The apparatus consists of a 26G needle positioned at the tip of a standard 2.5 µL micropipette. This allows for easy modification of the length of the needle tip, and permits rapid exchange of fresh sterile needles for different pathogens sequentially. We introduce this design and its advantages in performing intravitreal injections in the mouse eye, leading to the successful establishment of clinical endophthalmitis.

##  METHODS

### Maintenance and Housing of Mice

All mice were maintained at a temperature of about 20–25ºC with a relative humidity of about 30% to 70%, and 12-hr diurnal per day. Mice were caged according to the infection group with a maximum of six mice per autoclaved polycarbonate cage. The animals were acclimatized to the experimental conditions for at least five days before the start of the infection and checked by a veterinarian for their suitability for the study. The mice were also provided standard rodent pellets during the acclimatization period.

### Bacterial and Fungal Strains Used in the Study

For our study, we used *Staphylococcus aureus* and *P. aeruginosa *strains which were isolated from patients with bacterial endophthalmitis after appropriate consent. These strains were cultured on MHA (Mueller Hinton Agar) media and 1–2 colonies from 18-hr cultures were resuspended in Brain Heart Infusion (BHI) media; the concentration was set to achieve the final concentration of 10,000 CFU/µL. Similarly, *Candida albicans* and *A. flavus *strains were isolated from patients with fungal endophthalmitis. *Candida albicans* was grown on Sabouraud dextrose agar (SDA) containing 5% chloramphenicol. A single isolated colony from an 18-hr culture was resuspended in 1 × sterile phosphate buffered saline (PBS) and diluted to achieve the final concentration of 10,000 CFU/µL. In case of *A. flavus, *spores from the five-day old culture were resuspended in sterile 1 × PBS to achieve a final concentration of 15,000 spores/µL.

### Design of Intravitreal Injector

A 26-gauge needle of a 1 mL syringe (Dispo Van, Hindustan syringes and medical devices, Turbulin) was separated from the needle hub [Figure 1A] and inserted into a 1–10 µL tip (Eppendorf, Cat No: 0030000811) with the help of forceps as shown in Figure 1B. The snug fit of the needle was confirmed, and the external length of the needle was adjusted. This modified needle-injector tip was gas sterilized for further use. The intravitreal injections were then performed with a 2.5 µL pipette attached to the modified needle-injector tip using a tri-manual technique.

### Accuracy of Injection Quantity

A pilot study of 15 test samples was performed to measure the amount of solvent injected by our modified needle injector. We injected 15 separate aliquots of 1 µL of crystal violet dye dissolved in water onto a glass slide and immediately re-aspirated it to measure the amount of the injected material. This step was performed to ensure that the modification is able to inject an accurate and precise amount of solvent that was aspirated for injection.

### Intraocular Injections

All mice were anaesthetized with a combination of ketamine (35 mg/Kg) and xylazine (5 mg/Kg) by intramuscular route and topically anaesthetized with 0.5% proparacaine hydrochloride. Once the mouse was adequately anaesthetized until complete suppression of pedal reflexes, the pupil was dilated by 1% tropicamide (Ophtha pharma, India) to allow direct visualization of the needle when inserted inside the eye during injection. Both eyes were cleaned with betadine 10% solution (Win-Medicare, India) and wiped with cotton swab. The mouse was then laid down and with the help of a soft sponge of 1 cm height, so as to achieve tangential injection into the eye. The mouse's head was secured with the nondominant hand of the primary injector (ophthalmologist), and the tip of the 26G needle was placed at the limbus of the eye with the micropipette held in the dominant hand. With the needle in the bevel up position and at a 45º angle, the eye was punctured, ensuring that only the sharp tip of the needle (0.5 mm) was advanced into the eye [Figure 2A]. After the entry of the needle, allowance was made for some intraocular fluid to escape. This helped in lowering the eye pressure, thereby allowing complete retention of the subsequent injection of the suspension. The needle was then redirected posteriorly, tangential to the globe wall to reach the vitreous cavity without touching the crystalline lens. The direction and location of the needle was monitored through the microscope (ZEISS Stemi 508 Stereo Microscope, Germany). Once the needle tip was advanced up to the posterior vitreous, the secondary injector gently stabilized the pipette with one hand, and pushed the plunger of the pipette to inject 1 μL of the bacterial/fungal suspension into the vitreous cavity with the other hand. During this step, the needle tip was held stable within the eye by the primary injector. Complete emptying of the suspension was confirmed by direct observation of the Eppendorf tip. The tip of the needle was held inside the mouse eye for 2–3 s before withdrawing to avoid leakage during the injection process. Since one hand of the primary injector and both hands of the secondary injector were used, we call it a tri-manual technique. The treated eye was closed by a sterile dressing pad and the mice were placed in a separate cage until recovery from the anesthesia. If the lens or retina was damaged during injection, the mouse was not considered for further study. All procedures were performed by the same surgeon.

While the right eye was infected to cause endophthalmitis, the contralateral eye was injected either with 1 × PBS (surgical control), BHI (vehicle control) or was left without any injection (absolute control). The mice eyes were monitored throughout the course of infection (24 hr for bacterial, and 24 and 72 hr for fungal infection) using a slit-lamp (PSLAIA-11, Appasamy associates, Chennai, India) microscope [Figure 2B] and photographs were taken to document the disease severity and progression. A video demonstrating the preparation of the modified tip/needle injector and the intravitreal injection has been included [Video S1].

### Clinical Scoring

Prior to enucleation, eyes were clinically scored from 0 to +4 for anterior chamber inflammation, presence of red reflex, vitreous haze, and retinal clarity by an ophthalmologist who was masked to the type of injection in each mouse [Table 1].^[11]^ Anterior chamber was scored based on the number of cells and the intensity of flare and intensity of iris vessel dilation. Red reflex is the reddish-orange reflection of light from the retina during ophthalmoscopy which is present under normal conditions and progressively decreases with vitritis. Vitreous and retinal clarity is based on the visibility of the posterior pole and retinal details. The mean of these scores were calculated and plotted in a bar graph in prism software (version: 5.03, Insightful Science, California). Photographs were captured to visualize the disease progression. For clinical scoring, all values represent mean ± standard error of the mean (SEM) for triplicate samples per time point. Mann–Whitney U-test was used to statistically compare control and infected groups. A *P-*value ≤ 0.05 was considered statistically significant.

### Enucleation and Harvesting of Eyeballs

The mice were euthanized using carbon dioxide asphyxiation. The head of the mouse was held secure, and a fine tip forceps was used to induce proptosis in the eye [Figure 2C]. Once the forceps prongs were behind the globe, they were approximated to detach the eyeball along with the optic nerve [Figure 2D]. The harvested eyeball was immediately placed into a labelled tube, on ice.

### Viability Testing of Pathogens

The enucleated eyes from three biological replicates (three from surgical control and three from each infected set) were placed in a 2 mL Eppendorf tube individually with sterile 1X PBS and homogenized (EZ-lyser, Genetix Biotech Asia Pvt Ltd, India) immediately using sterile metal beads (3 mm). The 10 ${\;}^{-4}$ dilution of viable bacteria (*S. aureus* and *P. aeruginosa*) and the10 ${\;}^{-2}$ dilution of fungus (*A. flavus* and *C. albicans*) extracted from the infected eyes were then plated onto Mueller-Hinton Agar (MHA) or SDA media, respectively. The plate was checked for the growth of the respective organism after 24–48 hr of incubation.

### Histopathological Analysis

Half of the eyes of the euthanized animals from three biological replicates were immediately fixed in 10% buffered formalin (cat no-24008, Fisher Scientific) for 24 hr. Eyeballs were then placed in labelled cassettes and subjected to 6-hr schedule of an automatic tissue processor (Leica, TP 1020) used to completely dehydrate the specimen which was then followed by embedding in paraffin wax melted at 56ºC. The embedded tissue blocks were placed in a microtome (Leica, RM2125RTS) and sectioned down to 4 μm thickness to form ribbons of sections which were then floated out in a warm water bath maintained at 58ºC for 2 s. The sections were then carefully picked up on the glass slide using a filbert brush, which was gently pressed to remove air bubbles, drained upright, and dried thoroughly. The slides were then placed on a hot plate maintained at º for 30 min to melt the paraffin wax and improve adhesion of the tissues to the slide. These sections were immediately processed for Grams and Grocott's methenamine silver (GMS) staining for confirmation of bacterial and fungal infection respectively and to determine any retinal changes. The sections underwent sequential dehydration process with alcohol, were cleared with xylene, and finally mounted with DPX mount medium (Fine-chem limited, Mumbai).^[12]^

##  RESULTS

### Injection Technique

A total of 50 mice were injected using this novel technique. Two mice were excluded due to the formation of a cataract, presumably due to needle touch. Successful injection of the suspension could be performed in all 50 mice, however, clinical assessment was possible only in 48 mice. No technique or procedure-related complications were noted. Accuracy of the quantity of injection performed in a pilot of 15 samples was found to be accurate for all samples. During enucleation, all sites of injection were noted to be adequately sealed, with no rupture or expulsion of intraocular contents. Eyes from three biological replicates were used for all the experiments.

### Clinical Course of Endophthalmitis in a Mouse

There was a visible establishment of infection at 24 hr in bacterial/fungal-infected eyes when compared to controls [Figure 3]. The clinical scores of the eyes infected with *A. flavus* and *C. albicans* showed a significant increase in the disease progression both at 24 hr (*P*-value: < 0.00016; < 0.00001) and 72 hr (*P*-value: < 0.00001; < 0.0001) after the initial injection when compared to controls. Eyes infected with *S. aureus* and *P. aeruginosa* also exhibited significant increase in the disease progression at 24 hr (*P*-value: < 0.00001; < 0.00001) after the initial injection [Figure 4].

### Viability Testing

To confirm the purity of bacterial and fungal pathogens in the infected eye, diluted supernatant of homogenized eyeballs was plated onto MHA and SDA media, respectively. Pure isolated colonies of bacteria and fungi were grown on the media confirming the absence of any mixed infection [Figure 5].

### Histopathology Analysis

GMS staining for eyes infected with fungi (*A. flavus* and *C. albicans*) revealed the presence of fungal filaments in the retinal layers which were sequestered to the posterior lens surface. Gram-stained sections of the eyes infected with bacteria (*S. aureus *and* P. aeruginosa*) revealed their presence in the posterior region of the eye spreading across vitreous and retinal layers [Figure 6]. To assess the overall progress of endophthalmitis and compare the infected eyes with surgical control (PBS injected), the sections were stained with Haematoxylin and Eosin (H&E) which showed neutrophil infiltration within the posterior vitreous and dissolution of the retinal architecture in all infected eyes [Figure 7]. The H&E staining demonstrated the presence of significantly higher numbers of inflammatory cells when compared to control which has fewer cells probably due to the injection of PBS.

##  DISCUSSION

Successful animal models used for investigating ocular infections include the following animals: rabbits, guinea pigs, primates, swine, and mice.^[7]^ Mice serve as the best representable model of humans due to their genetic and pathogenetic similarity, despite having only 7 μL of vitreous.^[8,13]^The procedure of intravitreal injection and the quantification of infection parameters in a mouse endophthalmitis model has been described by several authors.^[11,14]^ A set of mouse model experiments can incur considerable costs to a research project, especially in developing countries. Intravitreal injections are required in small aliquots of microliters, necessitating the use of borosilicate microcapillary pipettes or Hamilton syringes of the size 50 μm for the endophthalmitis mouse models.^[6,11,15]^ Consequently, infection models require a fresh sterilized needle for each eye, amounting to large numbers of Hamilton syringes being utilized, adding to the cost of the experiment.

This study successfully demonstrates the use of a modified 26G needle attached to a laboratory micropipette for intravitreal injections in mice [Figure 8] to induce bacterial or fungal endophthalmitis using a tri-manual technique. It eliminates the need for the Hamilton syringe, and has several advantages. Firstly, the amount of the suspension in the injection is accurate, as confirmed by the pilot study measuring the accuracy of the injected test solvent. Secondly, it allows the use of a sharp disposable needle tip for each eye, and the preparation of one injection set takes less than 30 s. Thirdly, the short length of the needle (compared to the Hamilton needle) allows more precision while injecting. Although Hamilton syringes have an option to customize the needle length according to the requirements, the cost factor remains unsolved. In some cases, even diligent cleaning of the Hamilton syringe is inadequate. The tight tolerances between the glass and plunger will be compromised due to the deposits on the glass, resulting in a stiff plunger which cannot be interchanged. Using a fresh syringe for each injection per eye becomes necessary. Additionally, the use of fresh pipettes compounds the budget while trying to avoid cross-contamination while working on larger numbers of animals with different microorganisms.

One Hamilton syringe costs between USD 30 and 50 and may increase up to USD 100 depending on the customization of the needle size. Our modified injection pipette costs approximately 50–80 cents for a fully equipped unit per injection. Since the position of the needle tip within the eye is crucial, this was ensured under the magnification of the microscope using the tri-manual technique. Using this technique, we had a low and acceptable incidence of needle-related cataract formation, 2 mice out of 50 (4%).

Our study has limitations. The tri-manual technique requires skill and coordination among two injectors. Since the 26G needle is the smallest possible needle that can be used, it creates a relatively larger hole at the limbus, theoretically allowing egress of injected suspension. However, in our study, we noticed that the limbal hole did not cause any egress, and it healed within 6 hr of injection with no damage to the globe structures as observed by the ophthalmologist. Possibly the 45º-angled advancement of the needle and the decompression of the globe ensured that the injected fluid did not leak out despite using a 26G needle.

The second limitation is the accuracy of the quantity of injection, which is only tested for the intravitreal injection in our study. The maximum volume for intravitreal injections into the mouse eye is 1 μL.^[14]^ For mouse experiments beyond endophthalmitis, it is not known if any systematic error may occur with this new modified technique**. **An excessive volume would elevate the intraocular pressure which could result in globe rupture.

Finally, the enucleation of the mice eyes is a critical step, and needs to be performed gently to prevent retinal detachment or globe rupture, which might be falsely attributed to the injection technique. Another possible limitation could be that histopathological assessment was not performed on all of the controls (vehicle and absolute) and was done only for surgical controls, as the clinical assessment showed no difference among these controls. Although rare, we did see few inflammatory cells possibly due to injection of PBS, however, the count was very low, and clinically/statistically insignificant. Furthermore, during the processing of the tissue, a slight variation could have been induced.

In summary, our novel intravitreal injection technique leads to accurate and cost-effective delivery of the desired suspension in a mouse model of endophthalmitis.

## Ethical Considerations

C57BL/6 mice (6–8 weeks old, Sipra labs limited, Hyderabad, India) were used in this study. All animals were strictly maintained according to the guidelines of the Association for Research in Ophthalmology and Vision Research (ARVO) for the use of animals in ophthalmic and vision research and housed as per the Committee for the Purpose of Control and Supervision of Experiments on Animals (CPCSEA) guidelines in standard polycarbonate cages (six animals per cage). Protocols were meticulously designed to minimize animal suffering. All procedures were approved by the Institutional Animal Ethics Committee of Sipra Labs Limited (Protocol approval number: SLL/PCT/IAEC/20-21/C).

## Financial Support and Sponsorship

None.

## Conflicts of Interest

None.
